# Relationship between suppressor of cytokine signaling-3 gene polymorphism and growth pattern of adolescent idiopathic scoliosis

**DOI:** 10.1186/1748-7161-10-S1-O4

**Published:** 2015-01-19

**Authors:** Jun Qiao, Xusheng Qiu, Leilei Xu, Feng Zhu, Zezhang Zhu, Zhen Liu, Jun Jiang, Bangping Qian, Yong Qiu

**Affiliations:** 1 Spine Surgery, Affiliated Drum Tower Hospital of Nanjing University Medical School, Nanjing 210008, China

## Objective

To explore whether the suppressor of cytokine signaling-3 (SOCS3) gene polymorphisms are associated with the susceptibility, abnormal growth pattern of adolescent idiopathic scoliosis (AIS).

## Methods

398 AIS girls aged 10 to 18 years, and 367 age-matched healthy girls were recruited. Only patients who had Cobb angles larger than 20° were included in this study. Rs4969198 was selected as tagSNP to cover all of the related polymorphisms on SOCS3. Genotyping was performed using PCR-based Invader assay with the probe sets designed and synthesized by Third Wave. The genotyping results were read with an ABI PRISM7900HT sequence detection system (Applied Biosystems, Foster City, CA). A subgroup of 322 skeletally mature AIS patients who did not received bracing or any other conservative treatment previously were analyzed to define the contribution of rs4969168 on curve severity, body height, body weight and BMI.

## Results

Rs4969198 was successfully genotyped. No significant difference of genotype frequencies from the Hardy-Weinberg equilibrium (HWE) test was noted for the AIS patients or the normal controls. Neither the genotype nor the allele frequencies of rs49691968 were significantly different between the AIS patients and the normal controls. Rs4969168 was not found to be associated with the curve severity of scoliosis and body height. AIS patients with AA genotype had significantly higher body weight and BMI than the patients with AG and GG genotype (P=0.014)

## Conclusion

The SOCS3 gene polymorphisms are not associated with the occurrence of AIS, but the gene polymorphism (rs4969168) is associated with abnormal growth pattern of AIS, indicating that SOCS3 gene might be a disease-modifying gene of AIS.

